# Heterogeneous kinetics of AKT signaling in individual cells are accounted for by variable protein concentration

**DOI:** 10.3389/fphys.2012.00451

**Published:** 2012-11-28

**Authors:** René Meyer, Lorenza A. D'Alessandro, Sandip Kar, Bernhard Kramer, Bin She, Daniel Kaschek, Bettina Hahn, David Wrangborg, Johan Karlsson, Mats Kvarnström, Mats Jirstrand, Wolf-Dieter Lehmann, Jens Timmer, Thomas Höfer, Ursula Klingmüller

**Affiliations:** ^1^Division of Systems Biology of Signal Transduction, German Cancer Research Center (DKFZ), DKFZ-ZMBH AllianceHeidelberg, Germany; ^2^Division of Theoretical Systems Biology, German Cancer Research Center (DKFZ)Heidelberg, Germany; ^3^Bioquant Center, University of HeidelbergHeidelberg, Germany; ^4^Institute for Physics, University of FreiburgFreiburg, Germany; ^5^Molecular Structure Analysis, German Cancer Research Center (DKFZ)Heidelberg, Germany; ^6^Fraunhofer-Chalmers Research Centre for Industrial Mathematics (FCC)Göteborg, Sweden; ^7^Center for Systems Biology (ZBSA), University of FreiburgFreiburg, Germany; ^8^Freiburg Institute for Advanced Studies, University of FreiburgFreiburg, Germany; ^9^BIOSS Centre for Biological Signaling Studies, University of FreiburgFreiburg, Germany; ^10^Department of Clinical and Experimental Medicine, Linköping UniversityLinköping, Sweden

**Keywords:** mathematical modeling, HGF, PI3 kinase, AKT, single cell heterogeneity, live cell imaging, primary hepatocytes, hepatocellular carcinoma

## Abstract

In most solid cancers, cells harboring oncogenic mutations represent only a sub-fraction of the entire population. Within this sub-fraction the expression level of mutated proteins can vary significantly due to cellular variability limiting the efficiency of targeted therapy. To address the causes of the heterogeneity, we performed a systematic analysis of one of the most frequently mutated pathways in cancer cells, the phosphatidylinositol 3 kinase (PI3K) signaling pathway. Among others PI3K signaling is activated by the hepatocyte growth factor (HGF) that regulates proliferation of hepatocytes during liver regeneration but also fosters tumor cell proliferation. HGF-mediated responses of PI3K signaling were monitored both at the single cell and cell population level in primary mouse hepatocytes and in the hepatoma cell line Hepa1_6. Interestingly, we observed that the HGF-mediated AKT responses at the level of individual cells is rather heterogeneous. However, the overall average behavior of the single cells strongly resembled the dynamics of AKT activation determined at the cell population level. To gain insights into the molecular cause for the observed heterogeneous behavior of individual cells, we employed dynamic mathematical modeling in a stochastic framework. Our analysis demonstrated that intrinsic noise was not sufficient to explain the observed kinetic behavior, but rather the importance of extrinsic noise has to be considered. Thus, distinct from gene expression in the examined signaling pathway fluctuations of the reaction rates has only a minor impact whereas variability in the concentration of the various signaling components even in a clonal cell population is a key determinant for the kinetic behavior.

## Introduction

Cancer heterogeneity is considered a result of clonal instability, followed by clonal evolution (Campbell and Polyak, 2007; Marusyk and Polyak, 2010), as it has been shown in cultured cell lines (Odoux et al., 2008; Dalerba et al., 2011). It has been postulated that multi-lineage differentiation can contribute to tumor heterogeneity (Reya et al., 2001; Jordan et al., 2006; Dalerba et al., 2007), but this still remains very controversial (Shackleton et al., 2009) and might strongly depend on the cellular context. However, the heterogeneity of individual cells in a tumor is an extremely important issue since it can cause differential responses to treatment resulting in incomplete tumor regression and contributing to overall poor efficiency of therapy in hepatocellular carcinoma (HCC) patients (Unsal et al., 1994; Shachaf et al., 2004) and other cancers (Brognard et al., 2001).

Cells also harbor non-genetic sources of random variability that are likely to contribute to heterogeneous responses to therapy. Even in isogenic populations, cells die at very different time points after the administration of pro-apoptotic drugs, and a sizable fraction of cells usually survives treatment (Spencer et al., 2009). Such non-genetic cell-to-cell variability has been extensively studied in gene expression. Swain et al. (2002) have demonstrated that two identical genes in a bacterial cell are transcribed with different time-varying rates. Similarly, two alleles of the same gene in mammalian cells show random differences in transcription in the absence of allelic imprinting (Mariani et al., 2010). These data demonstrate that random fluctuations in the biochemical reactions involved in gene expression cause measurable differences in protein concentration in individual cells. Cell-to-cell variability in signal transduction is much less investigated. In analogy to transcription, the unavoidable rate fluctuations in molecular interactions, phosphorylation reactions etc., could cause variable signaling processing in individual cells. In analogy to transcription, this phenomenon will be referred to as “intrinsic noise” (Swain et al., 2002). Cell-to-cell differences in the concentrations of signaling proteins (receptors, kinases, phosphatases, adapters etc.) are another source of variability that will ultimately be due to gene-expression noise. Because this type of heterogeneity would be imposed by processes that are external to signal transduction, we refer to it as “extrinsic noise.” In terms of mathematical models of signal transduction, the distinction between the two kinds of noise is particularly clear. Intrinsic noise acts directly on the reaction rates itself whereas extrinsic noise acts on the parameters (especially the protein concentrations).

Clearly, the study of noise in signal transduction requires measurements in individual cells. To interpret such data in a system akin to signal transduction, the yeast cell cycle, Kar et al. (2009) suggested by means of model analysis that intrinsic noise contributes more than extrinsic noise sources. In a live cell imaging study of the mammalian antiviral response, intrinsic, and extrinsic noise contributions in the activation of the IRF-3/7 and NF-κB signaling pathways downstream of the viral sensor RIG-I were found to be both large and of comparable magnitude (Rand et al., 2012). By comparison, the extent of cell-to-cell heterogeneity in growth factor-mediated signaling in mammalian cells as well as the relative contributions of intrinsic and extrinsic noise has so far remained unclear.

**Figure 1:**
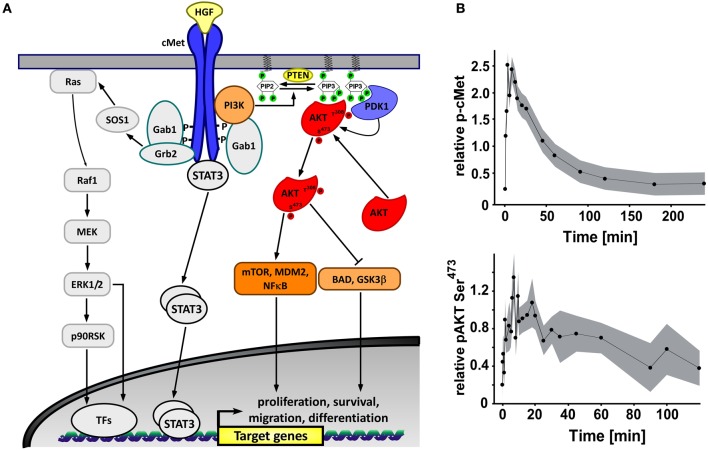
**Hepatocyte growth factor (HGF)-mediated signaling pathway. (A)** Graphical representation of the major signaling components of HGF-induced cellular responses with the cMet/PI3K arm highlighted in color. **(B)** Phosphorylation kinetics of the cMet receptor determined by quantitative immunoblotting (IB) and for AKT by quantitative protein array analysis in primary mouse hepatocytes stimulated with 40 ng/ml HGF. For the detection of cMet receptor phosphorylation immunoprecipitation with subsequent analysis by quantitative immunoblotting was employed that combines chemiluminescence with LumImager detection and quantification with the LumiAnalyst software. Measurements from triplicates of three-independent hepatocyte preparations have been merged on log scale assuming signal scaling between different gels. The merged signals are represented as parameters in a generalized least squares problem. Parameter estimates and one sigma confidence bounds are depicted as dots and error bands. For AKT the dots represent the scaled mean of the quantitative protein array results with one sigma confidence as error margins obtained from four different hepatocyte preparations.

A key growth factor that is not only essential for hepatocyte proliferation during normal liver formation and regeneration after injury, but also drives hepatic tumor cell proliferation (Patijn et al., 1998; Comoglio, 2001; Christensen et al., 2005; Michalopoulos, 2010; Joffre et al., 2011) is the hepatocyte growth factor (HGF). HGF binds to the receptor tyrosine kinase cMet, which activates receptor phosphorylation and subsequent activation of multiple signaling pathways including PI3 kinase signaling (Figure 1A). Among the HGF activated proteins, phosphatidylinositol 3 kinase (PI3K) and AKT play an important role in cell survival, growth, proliferation, angiogenesis, metabolism, and migration in normal and tumor context (Nicholson and Anderson, 2002; Manning and Cantley, 2007). It has been previously described that the different expression levels of PI3K signaling pathway components influence the pathway response to external stimuli (Yuan et al., 2011). By comparing single cell and population data in combination with mathematical modeling, we investigated if the heterogeneity is caused by stochastic fluctuations or extrinsic noise factors. To address this question, we monitored the dynamics of membrane recruitment of a mCherry-AKT fusion protein in primary mouse hepatocytes as well as in the hepatoma cell line Hepa1_6 and generated a population data-based deterministic ordinary differential equation (ODE) model. Based on the ODE model we performed stochastic analysis to investigate the variability derived by the different sources of noise at the single cell level. Our analysis demonstrated that the observed heterogeneity could not be explained by considering intrinsic stochastic fluctuations of proteins in individual cells alone, but rather there is a major contribution by extrinsic noise due to variations in total protein levels for all the involved signaling components.

## Results

### Population and single cell analysis of HGF signaling in primary mouse hepatocytes

To determine the dynamics of HGF signaling at the cell population level, primary mouse hepatocytes were stimulated with HGF and lysed at different time points. The activation of the HGF receptor cMet was determined by quantitative immunoblotting while AKT phosphorylation was quantified by quantitative protein array (Figure 1B). We observed a fast activation kinetic of cMet declining to the basal level after 180 min of HGF stimulation, while AKT phosphorylation shows a slower and sustained dynamics.

**Figure 2:**
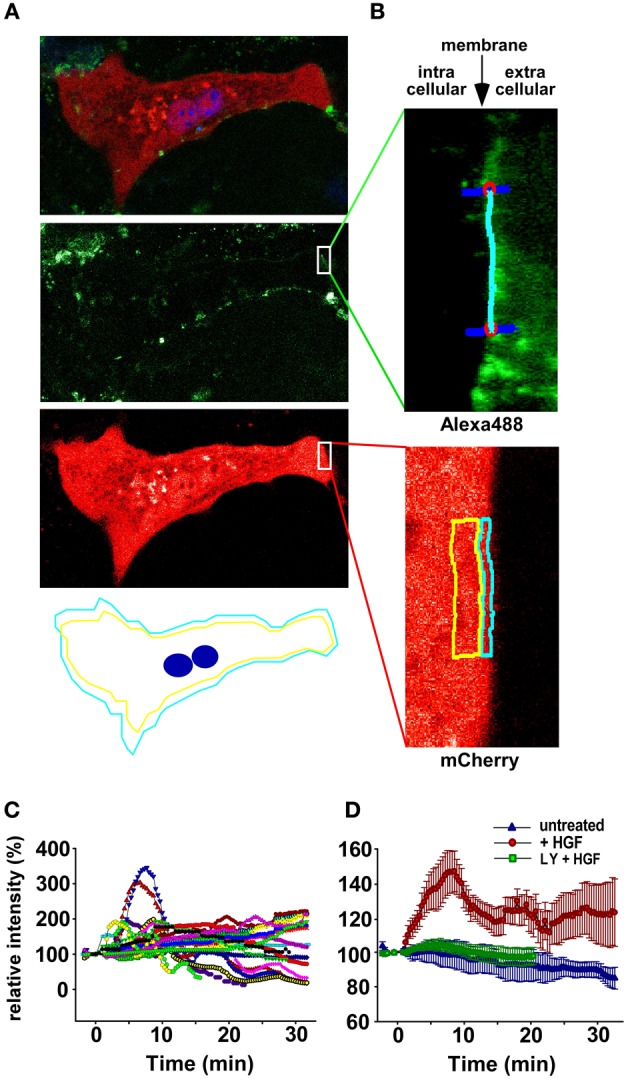
**Quantification of HGF-induced PI3K/AKT signaling at the single cell level. (A)** Confocal image of an individual mCherry-AKT transfected primary mouse hepatocyte is shown as overlay of the Hoechst, WGA-Alexa488, and mCherry-AKT signal with the signals from different channels in artificial-coloring. The graphical representation shows the tracked membrane signal in blue and the rim of the non-membrane cytoplasmic region in yellow with the localization of the two nuclei in the center. **(B)** Magnification of a subselection showing to the left of the tracked membrane section that is marked in blue the intracellular cytoplasmic space whereas to the right the bright green signal due to background staining of the WGA-Alexa488 visualizes the extracellular space. In the lower panel the quantification areas for mCherry-AKT intensity derived by the membrane tracking are depicted as blue (membrane associated) and yellow regions (intracellular reference area). **(C)** Signals from 25 individual single cell traces in response to 40 ng/ml HGF stimulation are represented in different colors. **(D)** Average of single cell traces are depicted for untreated controls (*n* = 12) in blue, after stimulation with 40 ng/ml HGF (*n* = 50) in red, and for cells pretreated with LY294002 for 30 min prior to HGF stimulation (*n* = 15) in green. Error bars represent the standard error of the mean.

In order to investigate if the cell population response is reflected at single cell level, fluorescently tagged AKT (Carpten et al., 2007; Landgraf et al., 2008) was employed to quantify the translocation of AKT to the plasma membrane and therefore its activation in individual cells. The mCherry-AKT localization was monitored by live cell imaging in transiently transfected primary mouse hepatocytes stimulated with HGF or left untreated. Localization of the fluorescently tagged AKT1 in unstimulated cells was similar as shown for different cell types in previous publications (Varnai and Balla, 2006; Carpten et al., 2007; Landgraf et al., 2008). In order to track the mCherry-AKT localization changes over time, the fluorescent signal was quantified within 5 pixels inside of the plasma membrane stained with WGA-Alexa488 as depicted in Figures 2A,B. The quantification of the track of 25 individual cells stimulated with HGF revealed a very heterogeneous single cell behavior (Figure 2C) from sharp transient peaks, double peaks, wavy behavior, or slow increase over the observation time of 30 min. By combining confocal imaging and TIRF microscopy, investigations of additional 50 cells from 10 independent primary mouse hepatocyte isolations confirmed the heterogeneous responses. To confirm that the observed mCherry-AKT localization changes are specifically triggered by HGF, cells were treated with PI3K inhibitor (LY294002) prior to HGF stimulation or left untreated, show unchanged mCherry-AKT localization as depicted for the average of the single cell traces (Figure 2D). Despite the heterogeneity of time courses of the HGF-induced AKT translocation to the cell membrane in individual hepatocytes, the average of the data obtained at the single cell level showed a remarkable similarity to the kinetics observed at the cell population level.

### Mathematical modeling of AKT signaling in primary mouse hepatocytes

**Figure 3:**
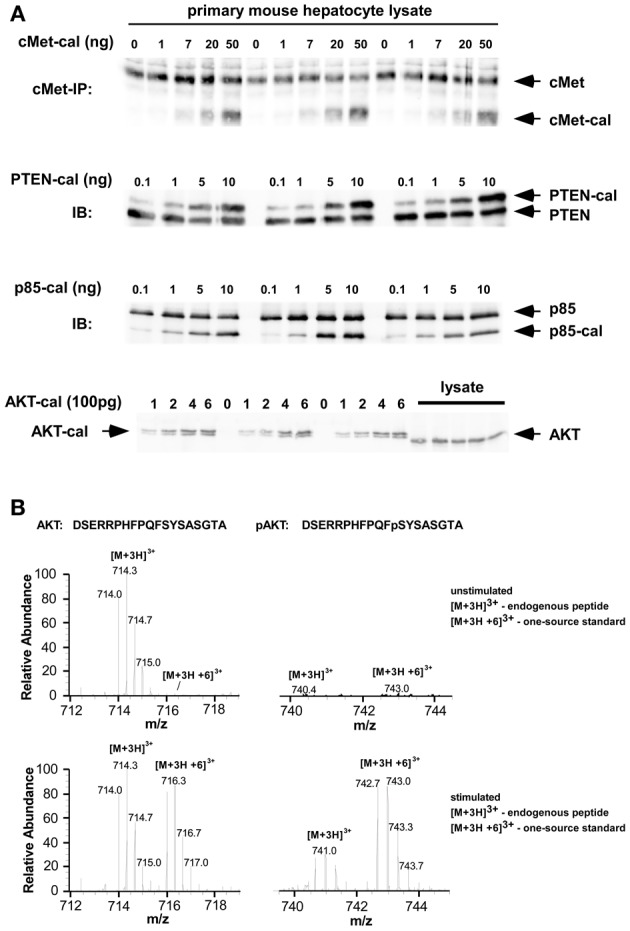
**Quantification of cMet and PI3K signaling components in primary mouse hepatocytes. (A)** Quantitative immunoblotting with known calibrator concentrations was used to estimate total number of molecules per cell and concentrations in untreated cell lysates. **(B)** Analysis of the degree of phosphorylation of AKT1 at Ser473 by mass spectrometry. Primary mouse hepatocytes were treated with 40 ng/ml HGF for 10 min or left untreated. Cells were lyzed, AKT1 was immunoprecipitated and in-gel digested. A one-source standard pair was labeled with ^13^C_6_-phenylalanine and added at 1:1 ratio to the digests prior to UPLC-MS/MS analysis. The figure shows the normalized mass spectra of the AKT1 peptides; upper panel: without stimulation, lower panel: after stimulation with HGF.

**Figure 4:**
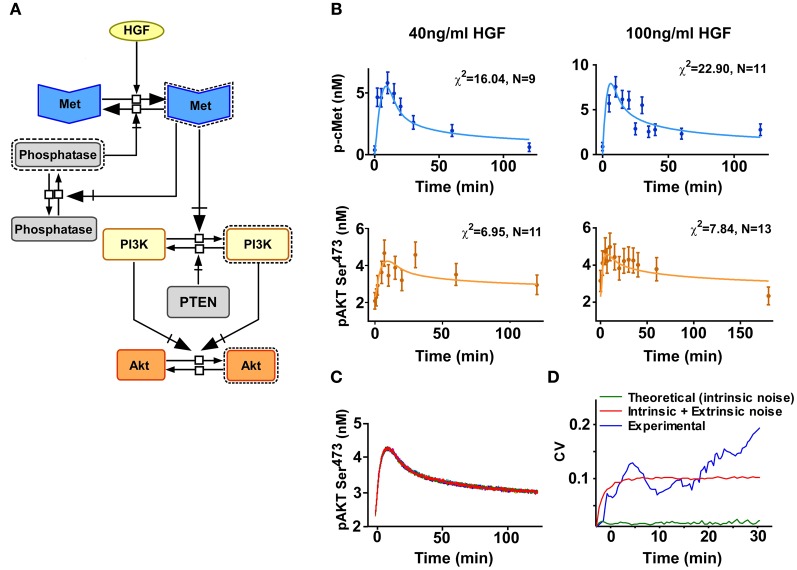
**Mathematical modeling of the cMet/PI3K signaling pathway. (A)** Schematic representation of the signaling pathway model generated with the Cell Designer Software. Species framed by dashed lines represent phosphorylated or activated forms. **(B)** Fits of time courses of cMet receptor and AKT phosphorylation in primary mouse hepatocytes stimulated with 40 ng/ml or 100 ng/ml HFG. Depicted as dots with standard deviation as error bar are the means of the indicated number of biological replicates. Model trajectories are depicted as lines and the corresponding Chi-square values are indicated. **(C)** Depicted in different colors are model simulations of AKT phosphorylation for 10 individual cells resulting from stochastic events. **(D)** The measured versus computed coefficient of variation (CV) for single cells over time are shown indicating the experimental fluctuations of mCherry-pAKT (blue line), theoretical intrinsic fluctuations of mCherry-pAKT (green line), and the corresponding combination of extrinsic and intrinsic fluctuation (red line).

To elucidate the mechanisms responsible for the observed heterogeneity, we developed a mathematical model of the PI3K/AKT signaling pathway activation. The model was initially formulated as set of deterministic ODEs for the concentrations of active cMet, PI3K, and AKT (Figure 4A). To constrain the model, the concentration of the key proteins of the pathway, cMet, the negative regulator PTEN, AKT, and the subunit p85 of PI3K protein, were determined by serial dilutions of recombinant protein standards in combination with quantitative immunoblotting (Figure 3A and Table 1). PI3K consists of two subunits, p110 and p85, and it has been shown that their level correlate (Ueki et al., 2002); therefore we quantified the p85 subunit to measure the abundance of PI3K. Additionally, the degree of AKT phosphorylation at 10 min post HGF stimulation was determined by quantitative mass spectrometry (Hahn et al., 2011) exemplarily shown in Figure 3B. All determined values and corresponding concentration ranges are summarized in Table 1. In addition to the above-listed proteins, the model includes a phosphatase for dephosphorylating cMet. Since we observed a basal level of AKT phosphorylation, we include in the model a direct activation of AKT by PI3K in a HGF-independent manner. In Figure 4B the best fit of the model to HGF-induced phosphorylation kinetics of cMet and AKT in a cell population is shown. The model equations and parameter values for the best fit obtained from 2500 fit sequences are given in Table 2. After fitting the experimental data, 50 of those 2500 fit sequences gave nearly identical sets of parameters.

**Table 1:** **Number of average molecules per cell and the phosphorylation degree of AKT at 10 min post HGF stimulation in primary mouse hepatocytes**.

	**Molecules per cell**	**Concentration (nM)**
cMet	92,000 ± 15,000	11.6
PTEN	32,000 ± 22,000	4.0
p85	38,000 ± 24,000	4.8
AKT	120,000 ± 60,000	15.1
mCherry-AKT	NA	NA
pAKT(Ser473)	23.0%	3.5
p-mCherry-AKT(Ser473)	NA	NA

Table 2**Equations and parameters for the primary mouse hepatocyte model**.

**Table:** 

**Primary mouse hepatocyte model: equations**	
$\frac{d\left[\text{pMet}\right]}{dt}=k_{\text{kMet}}\left(\left[{\text{Met}}_{\text{total}}\right]-\left[\text{pMet}\right]\right){\text{HGF}}_{\text{total}}-k_{\text{1Met}}\left[\text{pMet}\right]\left[{\text{Phos}}_{\text{active}}\right]$
$\frac{d\left[{\text{Phos}}_{\text{active}}\right]}{dt}=k_{\text{kPhos}}\left(\left[{\text{Phos}}_{\text{total}}\right]-\left[{\text{Phos}}_{\text{active}}\right]\right)\left[\text{pMet}\right]-k_{\text{1Phos}}\left[{\text{Phos}}_{\text{active}}\right]$
$\frac{d\left[\text{pMet-PI3K}\right]}{dt}=k_{\text{kPI3K}}\left(\left[{\text{PI3K}}_{\text{total}}\right]-\left[\text{pMet}-\text{PI3K}\right]\right)\left[\text{pMet}\right]-k_{\text{1PI3K}}\left[\text{pMet-PI3K}\right]\left[\text{PTEN}\right]$
$\frac{d\left[\text{pAkt}\right]}{dt}=k_{\text{kAkt}\_\text{back}}\left(\left[{\text{Akt}}_{\text{total}}\right]-\left[\text{pAkt}\right]\right)\left(\left[{\text{PI3K}}_{\text{total}}\right]-\left[\text{pMet-PI3K}\right]\right)+k_{\text{kAkt}}\left(\left[{\text{Akt}}_{\text{total}}\right]-\left[\text{pAkt}\right]\right)\left[\text{pMet}-\text{PI3K}\right]-k_{\text{1Akt}}\left[\text{pAkt}\right]$

**Table:** 

Primary mouse hepatocyte model: parameters	
**Parameter**	**Value**
*k*_kMet_	2.133E-01 nM^−1^ .min^−1^
*k*_1Met_	1.814 nM^−1^ .min^−1^
*k*_kPhos_	1.0E-04 nM^−1^ .min^−1^
*k*_1Phos_	1.0E-04 min^−1^
*k*_kPI3K_	1.63E-01 nM^−1^ .min^−1^
*k*_1PI3K_	3.399E-01 nM^−1^ .min^−1^
*k*_kAkt_ back_	1.316E-01 nM^−1^ .min^−1^
*k*_kAkt_	5.2E-01 nM^−1^ .min^−1^
*k*_1Akt_	3.476 min^−1^

To investigate whether intrinsic noise can account for the observed heterogeneity of AKT activation kinetics at the single cell level, we converted the deterministic model based on mass action kinetics (Table 2) into the corresponding stochastic model following the chemical master equation formalism (Kar et al., 2009). We simulated individual single cell traces (Figure 4C) using Gillespie's algorithm. The resulting intrinsic noise was too small to account for the experimentally observed single-cell behavior (Figure 4D). Therefore, we examined the contribution of extrinsic noise due to variable protein concentrations of the signaling components in individual cells. We distributed the total concentrations of all protein components in the model log-normally around the measured mean values with coefficient of variation (CV) of 0.15 (Niepel et al., 2009). The resulting cell-to-cell variability of AKT activation in the model was in the same range as the experimentally measured one (Figure 4D). This finding indicates that the heterogeneity of the total concentration of the signaling proteins in a heterogeneous population of primary mouse hepatocyte cells is the major contributor for the single-cell variability observed in mCherry-pAKT recruitment dynamics at the plasma membrane during HGF-mediated signaling.

### Population and single cell analysis in clonal cell populations

**Figure 5:**
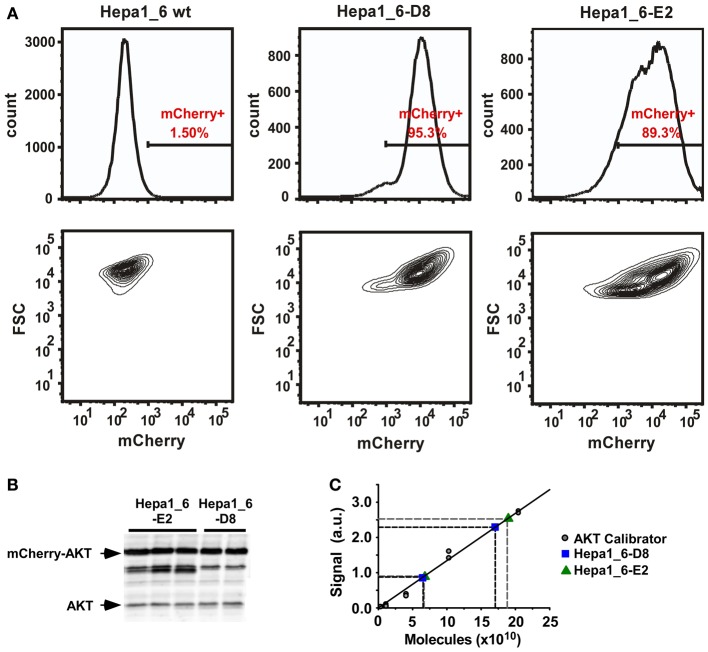
**Distribution and quantification of AKT expression levels in Hepa1_6 cell clones. (A)** FACS analysis of the distribution of mCherry-AKT expression in stable Hepa1_6 clones is shown in comparison to parental wild type Hepa1_6 cells. For the calculation of the coefficient of variation within the population the distribution in the Hepa1_6-E2 population (passage #12) is depicted on the right and the distribution in the Hepa1_6-D8 population (passage #11) is displayed in the middle. Their dependency on the cell size is shown in the corresponding lower panels. **(B + C)** Quantification of molecules per cell for mCherry-AKT and endogenous AKT in the stable Hepa1_6 clones D8 (35 μg total cell lysate) and E2 (20 μg of total cell lysate) are shown as determined by **(B)** quantitative immunoblotting and **(C)** linear regression from known AKT-calibrator concentrations analyzed on the same gel.

To rule out that the observed effects are due to variability introduced by transient transfection or result from hepatocytes derived from different regions in the liver, we generated stable Hepa1_6 cell clones expressing mCherry-AKT. Two clones, Hepa1_6-D8 and E2, were selected that showed high (Hepa1_6-E2) and intermediate (Hepa1_6-D8) mCherry-AKT expression levels based on flowcytometric analysis (Figure 5A). In addition, comparing by quantitative immunoblotting in both cell clones the concentration of mCherry-AKT and endogenous AKT, showed first of all a 1.6 fold higher endogenous AKT level in clone E2 compared to clone D8 and parental Hepa1_6 cells. For the mCherry-AKT expression in clone E2 was determined to be 2.0 fold higher than in clone D8. The mCherry-AKT expression was determined to be 4.3 fold (D8) and 5.5 fold (E2) higher than the endogenous AKT concentration in the respective clones (Figures 5B,C and Table 3).

**Table 3:** **Number of molecules per cell and concentrations of signaling components and phosphorylation degree of AKT and mCherry-AKT at 10 min HGF stimulation in the Hepa1_6 clones D8 and E2**.

	**Molecules per cell in Hepa1_6-D8**	**Concentration (nM)**	**Molecules per cell in Hepa1_6-E2**	**Concentration (nM)**
cMet	425,000 ± 71,000	65.2	378,000 ± 94,000	73.5
PTEN	47000 ± 25,000	27.3	220,000 ± 80,000	8.9
p85	775,000 ± 69,000	107.9	625,000 ± 47000	213.0
AKT	150,000 ± 112,000	94,7	549,000 ± 73,000	151.4
mCherry-AKT	1651,000 ± 533,000	405,5	2350,000 ± 582,000	825.7
pAKT(Ser473)	3.5%	3.3	2.5%	3.8
p-mCherry-AKT(Ser473)	0.7%	2.7	0.9%	7.6

**Figure 6:**
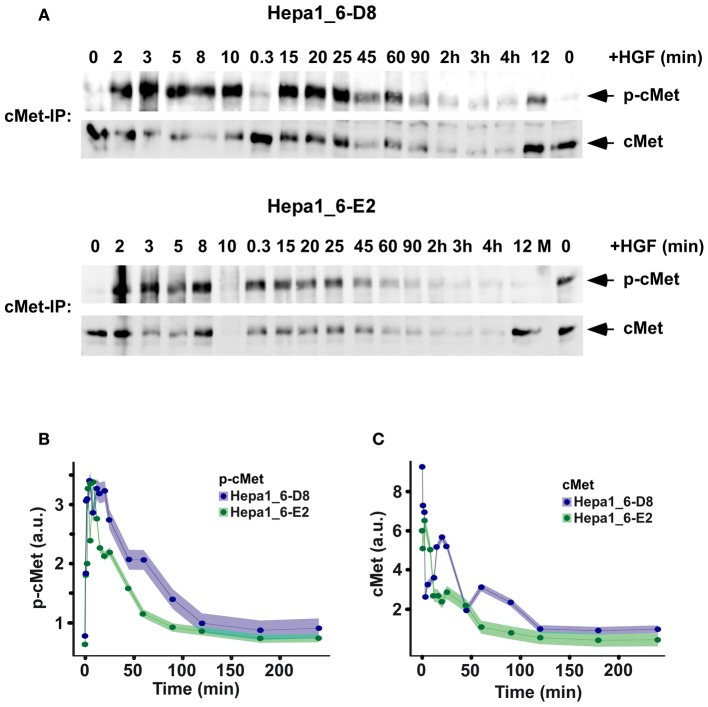
**Dynamics of cMet receptor phosphorylation and total receptor levels post HGF stimulation in Hepa1_6 cell clones stably expressing mCherry-AKT. (A)** Representative immunoblots for phosphorylation and total protein of the cMet receptor immunoprecipitated from lysates of Hepa1_6-D8 and Hepa1_6-E2 clone stimulated with 40 ng/ml HGF. **(B)** Experimental data indicated as dots show the mean of the kinetics of cMet receptor phosphorylation from three independent experiments and the shaded area indicates the standard deviation individually for both clones and in **(C)** the total cMet degradation dynamics is displayed.

**Figure 7:**
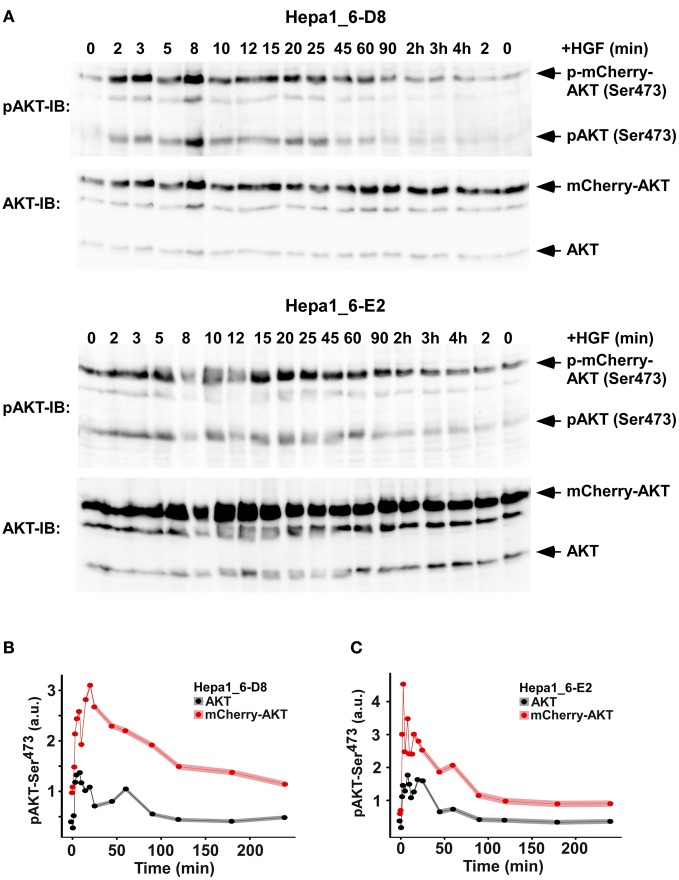
**Quantification of the phosphorylation dynamics and total protein levels of endogenous and mCherry-AKT post HGF stimulation in stable Hepa1_6 cell clones. (A)** Representative immunoblots for clone Hepa1_6-D8 and E2 detected with phosphor-Ser^473^ antibody and reprobing for total AKT are depicted. **(B + C)** Quantification of AKT phosphorylation dynamics from three-independent experiments are shown. The experimental data indicated by dots represents the mean (*N* = 3) and the shaded area indicates the standard deviation for **(B)** clone Hepa1_6-D8 and **(C)** clone Hepa1_6-E2.

To investigate if the overexpression of the mCherry-AKT construct is affecting the upstream signaling pathway, the time course of cMet phosphorylation and degradation dynamics in the two clones was compared (Figure 6A). The quantification showed that the receptor dynamics was not altered by the different exogenous AKT concentrations (Figure 6B). In order to determine if the mCherry-AKT followed the same dynamics as the endogenous one, their activation kinetics was directly compared by quantitative immunoblotting for both clones (Figure 7A). The quantification of mCherry-AKT phosphorylation dynamics was comparable to the endogenous AKT within each clone (Figures 7B,C). As expected, the amplitude of the mCherry-AKT phosphorylation signals was higher in both clones due to the higher concentration of the tagged AKT compared to the endogenous AKT. However, the total AKT levels remained constant over time independent of HGF stimulation. The similarity of the AKT phosphorylation dynamics independent of the different expression levels of endogenous and tagged AKT suggested that they both compete for the same interaction partners. In conclusion, we observed that there is no significant difference at the cell population level. Therefore, we investigated if there are major differences at the single cell level by monitoring the mCherry-AKT recruitment to the plasma membrane by live cell imaging as described for the primary mouse hepatocytes. The average of 10 single cell tracks for each clone depicted in Figures 9A,B showed a lower heterogeneity compared to one observed in the primary mouse hepatocytes.

### Mathematical modeling of AKT signaling in clonal cell populations

**Figure 8:**
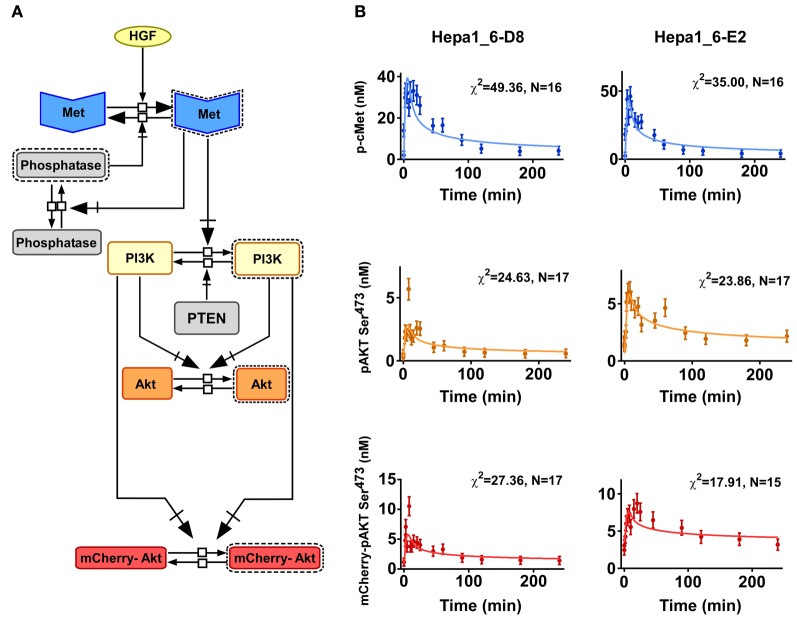
**Extended cMet/PI3K pathway model. (A)** Schematic representation of the model structure including mCherry-AKT. **(B)** Dynamics of the phosphorylation of cMet, endogenous AKT, and mCherry-AKT in the Hepa1_6-D8 and E2 clones stimulated with 40 ng/ml HGF. The means of the indicated number of biological replicates are represented as dots with the standard deviation as error bars. The model trajectories are depicted as lines and the corresponding Chi-square values are given.

**Figure 9:**
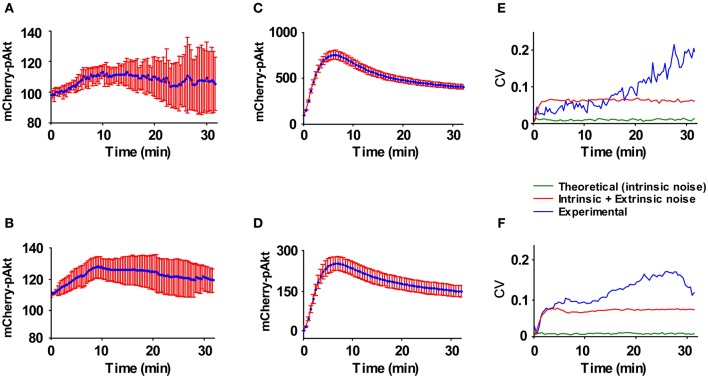
**Representation of experimental and model-derived CVs.** The average of the kinetics of mCherry-AKT membrane association in 10 individual cells for **(A)** the clone Hepa1_6-D8 and **(B)** clone Hepa1_6-E2 are shown with the standard error of the mean indicated for each time point. **(C + D)** The calculated dynamics for 10 simulated cells with extrinsic noise contribution as given by the parameters are shown. For the clones **(E)** Hepa1_6 D8 and **(F)** E2 the CV's for the experimental fluctuations in mCherry-pAKT (blue line), theoretical intrinsic fluctuations in mCherry-pAKT (green line), and the corresponding combination of extrinsic and intrinsic fluctuation (red line) are plotted.

As described for the primary mouse hepatocytes, we quantified the concentration of the pathway components in the Hepa1_6-D8 and E2 clones (Table 3). The degree of phosphorylation at 10 min post HGF stimulation in Hepa1_6 cell line was determined by mass spectrometry and calculated by comparative immunoblotting for the clones (Table 3). Additionally to the previous deterministic ODEs-based model generated for the primary mouse hepatocytes, the mCherry-AKT species was added to the model structure as depicted in Figure 8A. The new model included an HGF-independent activation of AKT both for the endogenous and for the mCherry-AKT. In a similar fashion to the primary mouse hepatocytes, the time resolved quantitative data generated for both clones were fitted to the new model (Figure 8B). The model reactions and the obtained parameter values are summarized in Table 4. Notably, the parameter sets were identical for both clones except for *k*_Akt_ and *k*_Aktc_. We implemented the same procedure as employed for the primary hepatocytes to transform the deterministic model to the corresponding stochastic model based on Gillespie's algorithm using chemical master equation formalism to simulate single cell traces for the clones. The intrinsic fluctuation calculated in the form of CV (green line) could not recapitulate the experimentally obtained CV (blue line) for both clones (Figures 9E,F). This was in agreement with the results obtained for the primary mouse hepatocytes, where intrinsic fluctuations could not account for the experimentally observed heterogeneity. Therefore, we investigated the effect of extrinsic fluctuation due to differences in protein concentrations by deriving the CV of the mCherry-AKT concentration in both clones by FACS analysis (Figure 5A). For simplicity the CV of all protein species were set to the measured CV of the mCherry-AKT of the corresponding clone, precisely CV of 0.137 for E2 and 0.096 for D8 clone. We simulated single cell traces for each clonal population by distributing the total protein concentrations of all the protein components in the model log-normally around the measured mean values and with the corresponding CV values obtained for the D8 and E2 clone (Figures 9C,D). The noise statistics (red line) calculated from these simulations resembled the heterogeneity observed in the experimental data (blue line) (Figures 9E,F) for the early response, suggesting that extrinsic fluctuations significantly contribute to the heterogeneity in particular during the early phase of signal transduction, whereas intrinsic fluctuations have only a minor impact. By comparing these results with the ones obtained in primary mouse hepatocytes, we confirmed that also in clonal populations extrinsic noise derived from variable expression levels of all considered proteins contributes most to the observed single cell heterogeneity of AKT response to HGF stimulation.

Table 4**Equations and parameters for the stable Hepa1_6 clone model**.

**Table:** 

**Hepa1_6 clone D8 and E2 model: equations**
$\frac{d\left[\text{pMet}\right]}{dt}=k_{\text{kMet}}\left(\left[{\text{Met}}_{\text{total}}\right]-\left[\text{pMet}\right]\right){\text{HGF}}_{\text{total}}-k_{\text{1Met}}\left[\text{pMet}\right]\left[{\text{Phos}}_{\text{active}}\right]$
$\frac{d\left[{\text{Phos}}_{\text{active}}\right]}{dt}=k_{\text{kPhos}}\left(\left[{\text{Phos}}_{\text{total}}\right]-\left[{\text{Phos}}_{\text{active}}\right]\right)\left[\text{pMet}\right]-k_{1\text{Phos}}\left[{\text{Phos}}_{\text{active}}\right]$
$\frac{d\left[\text{pMet}-\text{PI3K}\right]}{dt}=k_{\text{kPI3K}}\left(\left[{\text{PI3K}}_{\text{total}}\right]-\left[\text{pMet}-\text{PI3K}\right]\right)\left[\text{pMet}\right]-k_{\text{1PI3K}}\left[\text{pMet}-\text{PI3K}\right]\left[\text{PTEN}\right]$
$\frac{d\left[\text{pAkt}\right]}{dt}=k_{\text{kAkt}\_\text{back}}\left(\left[{\text{Akt}}_{\text{total}}\right]-\left[\text{pAkt}\right]\right)\left(\left[{\text{PI3K}}_{\text{total}}\right]-\left[\text{pMet}-\text{PI3K}\right]\right)+k_{\text{kAkt}}\left(\left[{\text{Akt}}_{\text{total}}\right]-\left[\text{pAkt}\right]\right)\left[\text{pMet}-\text{PI3K}\right]-k_{\text{1Akt}}\left[\text{pAkt}\right]$
$\frac{d\left[\text{pAktc}\right]}{dt}=k_{\text{kAktc}\_\text{back}}\left(\left[{\text{Aktc}}_{\text{total}}\right]-\left[\text{pAktc}\right]\right)\left(\left[{\text{PI3K}}_{\text{total}}\right]-\left[\text{pMet}-\text{PI3K}\right]\right)+k_{\text{kAktc}}\left(\left[{\text{Aktc}}_{\text{total}}\right]-\left[\text{pAktc}\right]\right)\left[\text{pMet}-\text{PI3K}\right]-k_{\text{1Aktc}}\left[\text{pAktc}\right]$

**Table:** 

**Hepa1_6 clone D8 and E2 model: parameters**	
**Parameter**	**Value**
*k*_kMet_	4.796E-01 nM^−1^ .min^−1^
*k*_1Met_	5.135E-01 nM^−1^ .min^−1^
*k*_kPhos_	1.0E-04 nM^−1^ .min^−1^
*k*_1Phos_	1.0E-04 min^−1^
*k*_kPI3K_	1.13E-02 nM^−1^ .min^−1^
*k*_1PI3K_	6.24E-02 nM^−1^ .min^−1^
*k*_kAkt_ back_	2.528E-03 nM^−1^ .min^−1^
*k*_kAktc_ back_	1.536E-02 nM^−1^ .min^−1^
*k*_1Akt_	126.3 min^−1^
*k*_1Aktc_	904.26 min^−1^
*k*_kAkt_ (for E2)	4.84E-02 nM^−1^ .min^−1^
*k*_kAkt_ (for D8)	1.637E-01 nM^−1^ .min^−1^
*k*_kAktc_ (for E2)	6.663E-02 nM^−1^ .min^−1^
*k*_kAktc_ (for D8)	5.224E-01 nM^−1^ .min^−1^

## Discussion

The response of cells to external cues is determined by the coordinated interaction of multiple signaling components. Heterogeneity in responses can arise from genetic variability, intrinsic stochastic fluctuations of reaction rates, and extrinsic noise in the form of variable protein expression levels even in clonal populations (Brock et al., 2009; Huang, 2009; Marusyk et al., 2012).

To determine the contribution of different sources of noise to the PI3K pathway activation upon HGF stimulation, we examined pathway activation at cell population and single cell level in two cellular model systems. In primary mouse hepatocytes major control mechanisms of signaling pathways are unperturbed. Therefore, this system closely represents the physiological situation and enables the analysis of molecular processes in a setting resembling the *in vivo* situation. We show that fluorescently labeled signaling components can be expressed in these cells albeit at very heterogenous levels. A major experimental limitation of the system is the variability of hepatocytes from preparation to preparation, the low transfection efficiency, and the uncontrollable expression levels. Although the HCC cell line Hepa1_6 harbors alterations in signaling pathways, it is a useful model system since cell clones can be selected that stably express labeled signaling proteins and thereby facilitate the examination of principle mechanisms.

As readout of PI3K pathway activation at the single cell level we monitored translocation of fluorescently tagged AKT to the plasma membrane. As previously demonstrated full-length AKT tagged at the N-terminus with green fluorescent protein (GFP) retains functionality comparable to the endogenous protein as demonstrated by its kinase activity and ligand-induced membrane translocation (Watton and Downward, 1999). In analogy to this construct we exchanged the GFP tag by a monomeric version (Campbell et al., 2002) of mCherry to avoid artifacts due to dimerization induced by the tag. We show that the mCherry-AKT fusion protein is phosphorylated in response to HGF stimulation and translocations to the membrane confirming functionality.

It has been shown that *in vivo* binding of AKT to PIP3 at the membrane is crucial for its activation by phosphorylation (Carpten et al., 2007; Landgraf et al., 2008; Gonzalez and McGraw, 2009). Experiments by Ding et al. showing that AKT can directly be phosphorylated by PDK1 without membrane recruitment if both are artificially co-localized by fusing each one to half of a fluorescent protein (Ding et al., 2010) suggest that localization to the membrane might merely serve as platform for AKT and PDK complex formation and thereby foster subsequent AKT phosphorylation. In line with previous reports (Coutant et al., 2002; Carpten et al., 2007; Landgraf et al., 2008; Gonzalez and McGraw, 2009), we show that membrane recruitment of mCherry-AKT is abolished in our experiments upon PI3K inhibition prior to HGF stimulation in line with the lack of phosphorylation at the population level confirming that membrane recruitment of mCherry-AKT serves as bona fide readout for PI3K pathway activation.

To disentangle the sources of noise contributing to the dynamics of PI3K pathway activation, we established a deterministic model based on time course data for phosphorylation of endogenous AKT. Subsequently, the parameters derived from this model were used for the stochastic model assuming that the parameters of the mCherry-AKT are similar to endogenous AKT. Stochastic models (Hayot and Jayaprakash, 2006; Lipniacki et al., 2006; Ashall et al., 2009) have been used to propose that cell-to-cell heterogeneity arises through intrinsic, stochastic, transcriptional variability, but this alone can not produce the highly different individual cell responses observed in our data. For cell cycle regulation the intrinsic fluctuations of the small number of mRNA molecules and overall low concentrations of expressed proteins are the major source of noise in the system (Kar et al., 2009). On the contrary, the single cell heterogeneity of growth factor signaling pathway activation, as shown here for HGF-mediated membrane recruitment and phosphorylation of AKT, cannot be explained by intrinsic noise alone suggesting only a minor impact of random fluctuations in reaction rates. Rather, the heterogeneity in pathway activation required the consideration of additional extrinsic noise pointing to the importance of variability in the concentration of pathway components in individual cells.

The expression level of pathway components in primary mouse hepatocytes probably due to low efficiency of transient transfection is very heterogeneous and correlates with highly variable pathway activation. By flow cytometry the CV for AKT expression was determined for the Hepa1_6 cell clones stably expressing mCherry-AKT underscoring the differences in overall expression levels and the range of expression. HGF-induced membrane recruitment of mCherry-AKT in those cell clones revealed cell-to-cell variability as already observed for the primary hepatocytes, but with overall less heterogeneity in the shape of the single tracks. This is probably due to the stable expression as compared to the transient transfection, which results in higher variability in the total protein level for each individual cell.

Independent of transient transfection or stable cell clones, our results show that extrinsic noise, in particular variability in the concentration of signaling components, overall contribute significantly to the observed noise at least for the initial kinetics of pathway activation. The later rise of the experimentally observed CV could be attributed to a number of factors: (1) induced mRNA expression and new protein synthesis, (2) a consequence of the experimental procedure with accumulated fluorescent bleaching due to the laser light, and hence (3) increased stress response and induced apoptosis as seen in some of the cells when imaged for more than 40 min. In the future, further fine-tuning of the modeling approach could be achieved by considering in addition mRNA concentrations, production and degradation rates, and correlation with the determined protein concentrations and their change over time depending on the treatment.

Single cell responses can be very diverse but still give a robust population response to physiological ligand concentrations for many different signaling pathways (Nelson et al., 2004; Turner et al., 2010) being able to trigger different specific responses depending on the cell context and temporal controls (Hoffmann et al., 2002; Ashall et al., 2009). Cell-to-cell variability within a cell population is one of the major causes of incomplete response of tumors to targeted therapy. Our results show that cell-to-cell variation in signal transduction is mainly due to extrinsic noise even in clonal populations. This knowledge could provide an important basis for the development of improved strategies for targeted tumor therapies in the future.

## Materials and methods

### Hepatocyte isolation and handling

The procedure for hepatocyte isolation and HGF stimulation has been previously established in our lab (Klingmuller et al., 2006; Castoldi et al., 2011; Huard et al., 2012). Primary mouse hepatocytes were isolated and subsequently cultivated for 4 h in adhesion medium in presence of 10% FCS and maintained over-night in the pre-starvation medium, that does not contain serum. The stimulation with 40 ng/ml of recombinant mouse HGF was performed after 6 h of starvation and cells lysed with NP-40 lysis buffer (1% NP-40, 150 mM NaCl, 20 mM Tris pH7.4, 10 mM NaF, 1 mM EDTA pH 8.0, 1 mM ZnCl_2_ pH4.0, 1 mM MgCl_2_, 1 mM Na_3_VO_4_, 10% glycerol) supplemented with aprotinin and AEBSF (Sigma-Aldrich) at different time points. For imaging purposes cells were seeded in 2-well Labtech chambers after collagen coating for 2 h at a density of 120.000 for primary hepatocytes and 80.000 for Hepa1_6 cells per ml per well. Cells were transfected in a total volume of 800 μl OptiMem using 6 μl Lipofectamine™ LTX and 4 μl Plus™ regency (Invitrogen), and 1 μg of Plasmid DNA. Transfection media was removed after 12 h and cells incubated in pre-starvation medium for at least 6 h. All biological assays and imaging where performed 24–48 h post transfection in starvation media.

### Quantitative immunoblotting

Serum-starved confluent Hepa1_6 cells or primary mouse hepatocytes were lysed at different time points after treatments and protein concentrations determined. To analyze c-Met activation an immunoprecipitation protocol using antibody Met(B-2) (Santa Cruz Biotechnologies, sc-8057) was established and the phosphorylation signal was detected using an anti-phosphotyrosine antibody 4G10 (Millipore, #05-1050). For all other components the total amount and the activation by phosphorylation was detected and quantified in immunoblots or protein array analysis using the following antibodies: pAKT(S473) #4058L, pAKT(T308) #4056S, and total AKT #9272S (Cell Signaling), total cMet (B-2) #sc-8057 (Santa Cruz), for pPTEN(Ser380/Thr382/383) #9554 and total PTEN #9552 (Cell Signaling), and total p85 #50-172-006 polyclonal serum (Upstate). Blots were developed using ECL advanced (GE Healthcare) with acquisition on an Image Quant LAS 4000 system and quantification with the Image Quant TL software (GE Healthcare). Repeated measurements have been merged on log scale assuming signal scaling between different gels. The merged signals are represented as parameters in a generalized least squares problem. Parameter estimates and 1 sigma confidence bounds are depicted as dots and error bands in Figures 1B, 6B,C, and 7B,C.

For absolute quantifications using dilution series of known concentrations of recombinant calibrator-proteins, SBP, or GST-tagged versions of the proteins PTEN, cMet, and p85 were cloned by PCR amplification from cDNA with introduction of appropriate restriction enzyme sites for ligation into the expression vectors. The cDNA for human PTEN was a kind gift from Alex Toker (Beth Israel Deaconess Medical Center, Boston, MA, USA), p85 from Michael D. Waterfield (University College London, UK), and cMet from George Vande Woude (Van Andel Research Institute, MI, USA). Calibrator-proteins were expressed in BL21 bacteria and purified using Avidin- or Gluthation-beads, respectively. The AKT calibrator was purchased as 6His-AKT (Millipore). SDS-Page with appropriate calibrator concentrations and biological replicates of the cellular lysates with subsequent quantitative immunoblotting was performed. Calibration curves were employed to determine the molecule number in the respective sample. Information on the used protein amount, number of the lysed cells, and the cell volume were used to estimate the molecules per cell and concentrations of the signaling components.

### Protein array analysis

To study the dynamic activation of the pathway components the hepatocytes were stimulated with HGF and time resolved data were generated by quantitative protein array analysis similar as previously published (Korf et al., 2008; Brase et al., 2011). Non-rabbit-derived antibodies were used for manufacturing the antibody arrays using Up05669 (Upstate), CS2967 (Cell Signaling), and sc-55523 (Santa Cruz) mouse antibody for AKT detection. The necessary pre-dilutions with PBS were tested, these are then diluted 1:1 with arraying buffer (Whatman). The spotting was performed with a sciFLEX-Arrayer–S5 (Scienion, Berlin) piezoelectric non-contact spotter on 16-pad nitrocellulose slides (Oncyte, Grace). Each antibody is spotted in 3 × 3 spots per pad. After spotting, the slides are stored at 4°C. For sample preparation fresh cell lysates are diluted with array buffer at a dilution in the range of 1:10 to 1:32. Depending on the protein of interest, the samples needed to be mildly denatured prior to dilution. The calibrator-proteins are treated similarly. Recombinant proteins were generated or are commercially available to be used as normalizers in immunoblotting and calibrators for the arrays containing a defined amount of the protein of interest with know phosphorylation degrees. The slides were blocked with LiCor Blocking Buffer for 2–6 h prior to incubation. Samples and calibrator-solutions were incubated on the slides shaking over night. All incubations were performed at 4°C. The slides were then washed with array buffer and incubated with specific rabbit-derived detection antibody [i.e., CS9272 (Cell Signaling), sc-1619, and sc-9272 (Santa Cruz) for AKT]. After removal of excess detection antibody, slides were washed again with array buffer, and then incubated with anti-rabbit-alexa680 coupled antibody. Afterwards, the slides were washed first with washing buffer and then with distilled water. The slides were then dried at room temperature in the dark and scanned using the LiCor Odyssey scanner (intensity 4–5, resolution 21 μm, high quality). The medians of the spot-intensities of the 9 spots per array pad and sample were quantified with the GenePix Pro Software. The calculation of the protein concentration was performed by an R-based custom made software (ProArray). The software uses the calibrator signals to estimate a multi-linear response matrix of each antibody with respect to the calibrator concentrations. This response matrix was inverted for assay signals in order to compute protein concentrations. Signal uncertainties were estimated based on the goodness of calibration. Subsequently, they were propagated to uncertainties of the computed concentrations.

### Microscopy

#### Laser scanning confocal microscopy

Live cell imaging was performed on a Zeiss LSM710 with an incubation chamber at 37°C and 5% CO_2_ using a 40× oil objective. Single transfected cells where imaged using Hoechst 34522 as nuclear DNA stain (blue), Wheat-germ-agglutinine-Alexa488 (WGA-Alexa488) (Invitrogen) as membrane stain (green) and the transfected mCherry-AKT (red). Time series imaging every 20–30 s or for 3D z-Stacks every minute where acquired in unstimulated 6 h starved cells and post HGF stimulation for at least 30 min or up to 2 h if applicable.

#### Cell tracking and mCherry-AKT quantification

Image analysis and quantification of mCherry-AKT membrane recruitment was done using the LSM-Zen2009 software, ImageJ, and a newly developed MatLab script for tracing the membrane stain in one channel over time with adjustments to cell movements and shape changes of the membrane (WGA-Alexa488) and quantifying the first 5 pixels inside the cell as membrane fraction in the second fluorescent channel (mCherry-AKT) with an further inside cytoplasmic region as reference. All values were normalized for bleaching during acquisition by the overall cell fluorescence.

#### Determination of the cell volume

Using the confocal Zeiss LSM710 with z-stack mode by consecutive imaging with adjacent not overlapping sections, the cell volume was determined by quantification of the cell area multiplied with the high of the confocal section summed up over all images.

#### Total internal reflection fluorescence (TIRF) microscopy

TIRF microscopy was performed at the Nikon Imaging Center of the University of Heidelberg with a Nikon Ti inverted microscope with perfect focus system for TIRF automated dual channel time-lapse imaging with laser lines of 488 and 561 nm. For detection an Andor iXon DU-897 Electron Multiplier CCD digital camera was used. Cells were imaged in 8-well labtech chamber slides in an environmental chamber from okolab, allowing for full temperature, CO_2_, and humidity control. The total intensity changes over time where quantified using ImageJ software representing the kinetic of mCherry-AKT at the membrane of the cells at the glass bottom due to the TIRF settings.

### Cloning/fluorescent tagging

The fluorescent tagging of AKT with mCherry was achieved by PCR amplification of mCherry-cDNA removing the stop-codon and replacing it with a short linker (Asp-Glu-Leu-Tyr-Lys-Gly-Thr-Gly-Ser-Ile) and the mouse AKT1 cDNA (Addgene #10841) sequence via an introduced BamHI restriction side in a similar fashion as described previously (Carpten et al., 2007; Landgraf et al., 2008).

#### Primers

mCherry-F-BglNhe: GAT AGA TCT GCT AGC ATG GTG AGC AAG GGC GAG GA

mCherry-R-KpnBam: GAT GGA TCC GGT ACC CTT GTA CAG CTC GTC CAT GC

mAKT1-F-BamHind: GAT GGA TCC AAG CTT ATG AAC GAC GTA GCC ATT GTG

mAKT1-R-EcoSal: GAT GTC GAC GAA TTC TCA GGC TGT GCC ACT GGC T

The mCherry-AKT fusion was initially cloned into the pENTR gateway entry vector via BglI/SalI into the BamHI/XhoI sites and subsequently cloned into the pMOWS-puro for stable cell line generation.

#### Generation and handling of stable cell line clones

Generation of retrovirally transduced stable clones was performed following standard protocols. Briefly, 10 μg of the final vector pMOWS-puro-mCherry-AKT was transfected into 293T-Phoenix-eco cells using CaCl_2_, the supernatant after 24 h was used for spin infection of 5 × 10^4^ Hepa1_6 cells and cells subjected to selection for 1 week with 2.5 μg/ml puromycin. Finally 500 cells were singled out and seeded on three 96-well plates to grow single cell clones in DMEM media with 5% FCS (Invitrogen), Pen/Strep, and Glutamax with additional 1.2 μg/ml puromycin supplementation for further cultivation. Finally, two single cell clones with different but stable mCherry-AKT expression levels were selected, namely clone Hepa1_6-D8 and E2.

### Mass spectrometry

For the analysis of the degree of phosphorylation of AKT1 at Ser473 one-source standard peptides labeled with ^13^C_6_-phenylalanine were used as previously described (Hahn et al., 2011). Primary mouse hepatocytes were treated with 40 ng/ml HGF or left untreated, respectively. Cells were lysed, AKT1 was immunoprecipitated and in-gel digested with AspN. For the cleavage peptides DSERRPHFPQFSYSASGTA und DSERRPHFPQFpSYSASGTA the one-source standard pair at 1:1 ratio was prepared and added to the digests prior to UPLC-MS/MS analysis.

### FACS

Flow cytometry analysis was performed on a LSR Fortessa equipped with five lasers (355, 405, 488, 561, and 633 nm) using FACS-tubes (Falcon, #352008). The mCherry-AKT was detected with the PI-channel settings with 0.4% compensation. The logarithmic transformed FACS values of mCherry-AKT intensity were used to calculate the CV of the protein expression of the population.

### Modeling

We constructed a mass action kinetics-based deterministic model for the simplified pathway scheme proposed in Figure 4A (for the primary hepatocytes) and Figure 9A (for the Hepa1_6 cell lines). The model equations are shown in Tables 2, 4, respectively. The parameter values in Tables 2, 4 are the best fits to the time course data for the phosphorylation of endogenous and exogenous AKT and the corresponding cMet phosphorylation obtained by the simulated annealing method. The tool used was Potterswheel (Potterswheel, Germany), a MATLAB toolbox for ODE-based chemical reaction simulations and fitting.

To account for the cell-to-cell variability in the AKT phosphorylation kinetics, we first investigated the effect of intrinsic noise theoretically. The deterministic models, formulated in terms of mass action kinetics, were converted into stochastic model using the chemical master equation formalism and simulated by using Gillespie's algorithm (Kar et al., 2009). The parameters values shown in the Tables 2, 4 were used for the stochastic simulations performed. The initial values for the concentration variables were calculated by using the total protein concentrations (Tables 1, 3) and the given parameter values. We simulated individual cell pAKT traces by drawing uniformly distributed random numbers required in stochastic simulation with a different starting seed of the random number generator. In a second step, we included “extrinsic” cell-to-cell variability by distributing the total protein concentrations according to experimental measurements of mean protein concentrations (Tables 1, 3) and coefficient of variations (CVs) (CV = 0.137 for E2 clone and CV = 0.096 for D8 clone). We drew random numbers from those log-normally distributed concentration distributions of the individual pathway components with different means and CV before the start of the stochastic simulation for an individual cell. In this way we simulated individual cells having different total concentration of the pathway components as well as intrinsic noise in the biochemical reactions.

### Conflict of interest statement

The authors declare that the research was conducted in the absence of any commercial or financial relationships that could be construed as a potential conflict of interest.

## Research Topic:

From structural to molecular systems biology: experimental and computational approaches to unravel mechanisms of kinase activity regulation in cancer and neurodegeneration

## Topic Editor:

Matteo Barberis, Humboldt University Berlin, Germany; Max Planck Institute for Molecular Genetics, Berlin, Germany
